# Supplementary material for: The effect of diabetes mellitus on COVID-19 mortality among patients in a tertiary-level hospital in Bandung, Indonesia
Source: PLoS One. 2023 Jun 15;18(6):e0286797. doi: 10.1371/journal.pone.0286797 (PMC10270330; doi:10.1371/journal.pone.0286797)
Supplement: Attachment — Submitted filename: Response to Reviewers.docx [file pone.0286797.s002.docx]

Manuscript ID PONE-D-22-31633

**The effect of diabetes mellitus on COVID-19 mortality among patients in a tertiary-level hospital in Bandung, Indonesia**

Dear Prof. Nosheen Nasir and Reviewers,

Thank you for the helpful comments on our manuscript. We have now addressed the issue raised and edited the manuscript accordingly.

Editor comments:

Please describe how your study differs from what is already reported about this association. State the strengths and limitations of your study in the discussion draft.

We acknowledge that other studies have investigated the association between diabetes mellitus (DM) and COVID-19 outcomes. However, there are additional strengths of our research besides this association. We added, “Compared to other studies investigating the association between DM and COVID-19 outcomes, this study provides more descriptive information about the laboratory characteristics of COVID-19 patients with DM in Indonesia, which could offer important knowledge for clinicians, especially for treatment considerations. Our study also only included patients with rRT-PCR results to minimize misclassification bias. Moreover, the sample size of our study is relatively large.” (Lines 275-280).

We also added, “Our study has limitations; firstly, the data used in this study were retrieved during the first COVID-19 wave in Indonesia. Therefore, even though it gives valuable insight into how DM affects COVID-19 patients, vaccination and the spread of infection later may change the clinical characteristics of COVID-19 patients with DM. Secondly, ...” (Lines 282-285).

Journal requirements:

1. Please ensure that your manuscript meets PLOS ONE’s style requirements, including those for file naming.

We have ensured that our manuscript meets the requirements.

2. In your Data Availability statement, you have not specified where the minimal data set underlying the results described in your manuscript can be found. PLOS defines a study’s minimal data set as the underlying data used to reach the conclusions drawn in the manuscript and any additional data required to replicate the reported study findings in their entirety. All PLOS journals require that the minimal data set be made fully available.

We have uploaded the minimal data set as a Supporting Information file.

The feedback is highly valuable, and we are grateful for your constructive input on our manuscript. Considering the raised concerns, we believe the paper has now improved.

On behalf of all authors,

Raspati Cundarani Koesoemadinata, MD, MSc

Research Center for Care and Control of Infectious Disease

Universitas Padjadjaran, Bandung, Indonesia
